# Structural and functional characterization of the cardiac mitochondria-associated reticular membranes in the *ob/ob* mouse model

**DOI:** 10.1016/j.jmccpl.2025.100453

**Published:** 2025-05-10

**Authors:** Hala Guedouari, Maya Dia, Juliette Geoffray, Camille Brun, Florentin Moulin, Lucas Givre, Lucid Belmudes, Christelle Leon, Stephanie Chanon, Jingwei Ji-Cao, Christophe Chouabe, Sylvie Ducreux, Claire Crola Da Silva, Ludovic Gomez, Yohann Couté, Helene Thibault, Jennifer Rieusset, Melanie Paillard

**Affiliations:** a University Claude Bernard Lyon1, CarMeN Laboratory- IRIS Team, INSERM, INRAE, 69500 Bron, France; b Laboratory of Experimental and Clinical Pharmacology, Faculty of Sciences, Lebanese University, Beirut, Lebanon; c Univ. Grenoble Alpes, INSERM, CEA, UA13 BGE, CNRS, FR2048, 38000 Grenoble, France; d University Claude Bernard Lyon1, CarMeN Laboratory– MERISM team, INSERM, INRAE, 69921 Oullins, France; e Hospices Civils de Lyon, 69500 Bron, France

**Keywords:** SR-mitochondria coupling, MERCs, Database, Diabetic cardiopathy, Mitochondrial calcium uniporter, ERMCs

## Abstract

Type 2 diabetes (T2D) and obesity strongly lead to diabetic cardiomyopathy (DCM). The involvement of mitochondria-associated reticular membranes (MAMs), a signaling hub in the cardiomyocyte, starts to be demonstrated in T2D-related metabolic disorders. We recently discovered a cardiac MAM Ca^2+^ uncoupling in a high-fat high-sucrose diet (HFHSD)-induced mouse model of DCM. To better determine the role of MAMs in the progression of DCM, we here aimed to characterize the proteomic composition and function of the cardiac MAMs of another obesogenic T2D mouse model, the leptin-deficient *ob/ob* mouse.

12-week old male C57Bl6-N *ob/ob* mice displayed strain rate dysfunction and concentric remodeling, while no change was observed in fractional shortening or diastolic function. Increased lipid deposition but no fibrosis was measured in the *ob/ob* heart compared to WT. Electron microscopy analysis revealed that cardiac MAM length and width were similar between both groups. A trend towards an increased MAM protein content was measured in the *ob/ob* heart. MAM proteome analyses showed mainly increased processes in *ob/ob* hearts: cellular response to stress, lipid metabolism, ion transport and membrane organization. Functionally, MAM-driven Ca^2+^ fluxes were unchanged but hypoxic stress induced a cell death increase in the *ob/ob* cardiomyocyte. Mitochondrial respiration, cardiomyocyte shortening, ATP and ROS content were similar between groups.

To conclude, at that age, while being strongly hyperglycemic and insulin-resistant, the *ob/ob* mouse model rather displays a modest DCM without strong changes in MAMs: preserved structural and functional MAM Ca^2+^ coupling but increased response to stress.

## Highlights

• 12-week-old ob/ob mice develop strain rate dysfunction and concentric remodeling
• The obesogenic and T2D ob/ob mouse does not display metabolic HFpEF at 12 weeks old
• No structural or functional change in SR-mito Ca^2+^ coupling of ob/ob cardiomyocytes
• Increased proteins of lipid metabolism and response to stress in cardiac ob/ob MAMs
• Increased hypoxia-induced cell death in ob/ob cardiomyocytes

## Graphical abstract

**Figure:**
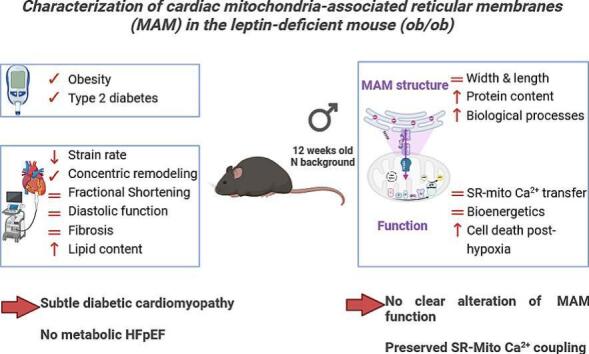

## 1 Introduction

The prevalence of type 2 diabetes (T2D) and obesity is dramatically increasing in the world [1]. By 2045, 10.9 % of people worldwide are expected to be affected by T2D [2]. Diabetic cardiomyopathy (DCM) is a chronic complication of T2D characterized by abnormal myocardial structure and function [[3], [4], [5]]. Myocardial remodeling, together with cardiac hypertrophy and diastolic/systolic dysfunctions are the hallmarks of DCM, a main contributor to heart failure (HF) in diabetic patients without any other cardiovascular risk factors or existing coronaropathy [6,7]. Among the different types of HF, T2D patients often evolve towards heart failure with preserved ejection fraction (HFpEF), leading to the so-called and most prevalent phenogroup of HFpEF, the cardiometabolic HFpEF [8]. Recently, gliflozins and GLP-1 agonists have shown a cardioprotective effect in cardiometabolic HFpEF, but still with a high residual risk [9,10]. Therefore, more research is needed to identify specific therapeutic targets in the progression of DCM.

Within the cardiomyocyte, mitochondria and sarcoplasmic reticulum (SR) interact at contact points to form microdomains known as mitochondria-associated reticular membranes (MAMs). These contact sites between SR and mitochondria play a relevant role inside the cardiomyocyte, notably in key functions such as Ca^2+^ transfer, lipid metabolism, autophagy, metabolic coupling and organelle dynamics [11], highlighting MAMs as a crucial signaling hub. Several studies have reported that abnormal structure or function of MAMs is linked to a high prevalence of cardiovascular diseases such as myocardial hypertrophy and heart failure [12]. Wu et al. demonstrated an increased MAM formation contributing to mitochondrial Ca^2+^ overload-driven cell death in a mouse model of type 1 diabetes-induced DCM with features of heart failure with reduced ejection fraction (HFrEF) [13]. On the other hand, we reported a reduced SR-mitochondrial Ca^2+^ coupling as an early trigger of reduced mitochondrial energetics in DCM using a high-fat high-sucrose (HFHSD)-induced mouse model with early symptoms of HFpEF, i.e. cardiac hypertrophy, diastolic dysfunction and reduced strain rate [14]. Interestingly, a structural SR-mitochondria Ca^2+^ uncoupling was demonstrated in hearts from T2D patients evolving towards cardiometabolic HFpEF [15]. Altogether, these studies support a leading role for the SR-mitochondria Ca^2+^ coupling in DCM. However, whether the SR-mitochondria Ca^2+^ uncoupling is a key trigger of all types of DCM or more specifically of cardiometabolic HFpEF remains unknown. Further investigations are needed, notably at the protein level.

Few proteomic analyses of MAMs have been conducted and were mainly assessed in brain in the context of diabetes or neurodegeneration [[16], [17], [18]], in liver [19] or in cells following virus infection [20,21]. Ma et al. demonstrated reduced endoplasmic reticulum-mitochondria interactions in the long-term diabetic mouse brain from the leptin receptor-deficient mouse (*db/db*), with altered MAM functions in cell proliferation, survival and inflammation responses [16]. To our knowledge, until now, there was no extensive report of the cardiac MAM proteome, except our recently published succinct description of the cardiac MAM proteome in the HFHSD mouse [14]. Therefore, an exhaustive mass spectrometry (MS)-based description and characterization of the mouse cardiac MAM proteome remains to be performed.

Based on this evidence, we investigated the role of the SR-mitochondrial Ca^2+^ coupling in a genetic mouse model of obesity and T2D, the leptin-deficient *ob/ob* mouse [8,22,23]. MS-based quantitative analysis of enriched cardiac MAMs, coupled with compositional and functional analysis was carried out to characterize the cardiac MAM proteome and function in the *ob/ob* model. Relevant biological pathways were further investigated by experimental validation on isolated adult mouse cardiomyocytes from *ob/ob* mice.

## 2 Material and methods

### 2.1 Animal studies

All animal procedures performed conform to the guidelines from Directive 2010/63/EU of the European Parliament on the protection of animals used for scientific purposes. Animal protocols used in this study were approved by the institutional animal research committee from Université Claude Bernard Lyon 1 (#15627–2,018,062,118,508,398; LYONSUD-2012-002). 12-week-old male B6·V-Lepob/OlaHsd (*ob/ob*) mice and their corresponding WT (C57BL/6JOlaHsd) mice were obtained from ENVIGO and are both on a N background, i.e. no mutation of the nicotinamide nucleotide transhydrogenase. Mice were housed as a group of 4 and fed ad libitum. A total of 66 mice was used in this study. All mice were sacrificed by cervical dislocation at fed state, unless specified differently. Cardiac insulin signaling was assessed in vivo by intraperitoneal injection of insulin (10 mU/g) in 6 h-fasted mice: 15 min later, mice were euthanized by cervical dislocation for quick removal of the heart and freezing in liquid nitrogen, finally stored at −80 °C until immunoblotting of phospho-AKT (1/1000; Cell Signaling 4060) and AKT (1/1000; Cell Signaling 4691) in 50 μg of total heart homogenates. Results are reported according to the ARRIVE guidelines 2.0 [24] (Supplemental Table 1).

### 2.2 Echocardiography

Systolic function was measured under light anesthesia (ketamine 80 mg/kg, IP), with a digital ultrasound system (Vivid 7, GE Medical Systems) and a 13-MHz linear-array transducer; while diastolic function was assessed on the Vevo 3100 imaging system using a 40-MHz linear probe (VisualSonics) under 2–3 % isoflurane anesthesia, as previously described [14].

### 2.3 Electron microscopy

For the ultrastructural study, isolated cardiomyocytes in-suspension were fixed with 2 % glutaraldehyde (EMS) in 0.1 M sodium cacodylate (pH 7.4) buffer. After washing three times in 0.2 M sodium cacodylate buffer, cell cultures were post-fixed with 1 % aqueous osmium tetroxide (EMS) for 1 h, dehydrated in a graded ethanol series at room temperature and embedded in Epon. After polymerization, ultrathin sections (100 nm) were cut on a UC7 (Leica) ultramicrotome and collected on 200 mesh grids. Sections were stained with uranyl acetate and lead citrate (EMS) before observations on a Jeol 1400JEM (Tokyo, Japan) transmission electron microscope equipped with an Orius 600 camera and Digital Micrograph. This microscope is located at the CIQLE platform (Centre d'Imagerie Quantitative Lyon Est, France). Interfaces between SR and mitochondria were blindly analyzed using a custom Image J plugin/macro, as previously published [25].

### 2.4 Histology

Hearts were fixed with 4 % paraformaldehyde and then paraffin-embedded to perform a Sirius Red staining for fibrosis. Sections were observed using the Zeiss AxioScan Z1 slide scanner. Images were blindly analyzed using a homemade macro on ImageJ. Analysis was performed using a custom-written Fiji macro.

### 2.5 Triglycerides

The total triglyceride content was evaluated from mouse heart lysates using an enzymatic kit (Biolabo, Maizy, France).

### 2.6 Heart subcellular fractionation to obtain MAMs

Cardiac MAMs were isolated following the protocol for liver MAMs from Wieckowski et al. [26], except for the first homogenization which was done using a Teflon-glass grinder (15–20 strokes, 300 rpm) as previously described [14]. Atria were removed and heart tissues were processed according to the protocol. Protein content was determined by the Lowry DC Protein Assay (Biorad) for each fraction, and fractions were finally frozen at −80 °C until further processing. MAM purity was checked by immunoblotting: Grp75 (sc133137, 1/1000), VDAC (ab14734, 1/1000), Cytochrome c (sc131156, 1/1000), Tubulin (sc5286, 1/1000).

### 2.7 Mass spectrometry (MS)-based proteomic analyses

MAM proteins purified from heart of WT and *ob/ob* mice (4 biological replicates for each) were solubilized in Laemmli buffer and heated for 10 min at 95 °C. They were then stacked in a single band at the top of a 4–12 % NuPAGE gel (Invitrogen), stained with Coomassie blue R-250 (Bio-Rad) before in-gel digestion using modified trypsin (Promega, sequencing grade) as previously described [27]. The resulting peptides were analyzed by online nanoliquid chromatography coupled to MS/MS (Ultimate 3000 and LTQ-Orbitrap Velos Pro, Thermo Fisher Scientific) using a 120-min gradient. For this purpose, the peptides were sampled on a precolumn (300 μm × 5 mm PepMap C18, Thermo Scientific) and separated in a 75 μm × 250 mm C18 column (PepMap, 3 μm, Thermo Fisher Scientific). The MS and MS/MS data were acquired using Xcalibur (Thermo Fisher Scientific).

Peptides and proteins were identified by Mascot (version 2.6.0, Matrix Science) through concomitant searches against the UniProt database (*Mus musculus* taxonomy, April 2020 version), a homemade database containing the sequences of classical contaminant proteins found in proteomic analyses (such as human keratins, trypsin…), and the corresponding reversed databases. Trypsin/P was chosen as the enzyme and two missed cleavages were allowed. Precursor and fragment mass error tolerances were set at 10 and 0.6 Da, respectively. Peptide modifications allowed during the search were: Carbamidomethyl (C, fixed), Acetyl (Protein N-term, variable) and Oxidation (M, variable). The Proline software (version 2.0) [28] was used for the compilation, grouping, and filtering of the results (conservation of rank 1 peptide-spectrum matches, peptide length ≥ 6 amino acids, false discovery rate of peptide-spectrum-match identifications <1 % as calculated on scores by employing the reverse database strategy, and minimum of one specific peptide per identified protein group). MS data have been deposited to the ProteomeXchange Consortium via the PRIDE partner repository [29] with the dataset identifier PXD054672. Proline was then used to perform a MS1 label-free quantification of the identified protein groups based on specific peptides.

Statistical analysis was performed using the ProStaR software [30]. Proteins identified in the contaminant database, proteins identified by MS/MS in less than two replicates of one condition, and proteins quantified in less than four replicates of one condition were discarded. After log2 transformation, abundance values were normalized using the variance stabilizing normalization (vsn) method, before missing value imputation (SLSA algorithm for partially observed values in the condition and DetQuantile algorithm for totally absent values in the condition). Statistical testing was conducted with limma, whereby differentially expressed proteins were selected using a Fold Change cut-off of 1.75 and a *p*-value cut-off of 0.05, allowing to reach a false discovery rate inferior to 5 % according to the Benjamini-Hochberg estimator.

Compartmental distribution was assessed manually for each protein using the uniprot.org website, whereas for protein molecular functions and biological processes, Panther software (pantherdb.org) was used (**Supplemental Table 2**).

### 2.8 Cardiomyocyte isolation and calcium imaging

One week after an intramyocardial injection of an adenovirus encoding the FRET-based mitochondrial Ca^2+^ sensor 4mtD3cpv, as previously described [14], cardiomyocytes were isolated by collagenase digestion based on O’Connell's protocol [31]. [Ca^2+^]_m_ levels were measured using a widefield Leica DMI6000B microscope equipped with a 40× objective and an Orca-Flash4.0 digital camera (HAMAMATSU), by calculating the YFP/CFP ratio. Histamine (10 mM) was used to follow the Ca^2+^ transfer into mitochondria after IP3R stimulation, in CCB (140 mM NaCl, 5 mM KCl, 1 mM MgCl_2_, 10 mM HEPES, 2 mM CaCl_2_ with 10 mM glucose) at 37 °C.

### 2.9 Mitochondrial calcium uptake

Cardiac mitochondria were isolated and Ca^2+^ clearance assays were performed as previously described [14].

### 2.10 Patch clamp electrophysiology

Current recordings were made at room temperature under voltage clamp using the whole-cell configuration of the patch-clamp technique. Command voltage and data acquisition were performed with pClamp software (Axon Instruments, Foster City, CA, USA). L-type Ca^2+^ currents were evoked every 10 s by 250-ms voltage steps spaced 10 mV apart and varying between −90 to 60 mV and were measured as the difference between the peak inward current and the current at the end of the pulse. The holding potential was kept at −80 mV. Membrane capacitance was systematically measured and calculated by analyzing the capacitive surge produced by a small voltage step as previously described [32]. Current traces were uncorrected from the leak and normalized to the capacitance. The external solution contained (in mM): 136 TEACl, 2 MgCl_2_, 1.8 CaCl_2_, 5 4-aminopyridine, 10 glucose, 10 Hepes, adjusted to pH 7.4 with TEAOH. The internal solution contained (in mM): 140 CsCl, 1 MgCl_2_, 3 MgATP, 10 EGTA, 10 Hepes, adjusted to pH 7.2 with CsOH.

### 2.11 Field-stimulated cytosolic Ca^2+^ transients and cell shortening

Cytosolic Ca^2+^ transients were measured on 1 μM Fluo-4 loaded cardiomyocytes (Thermo Fisher F14201: 30 min at 37 °C) with a laser scanning confocal microscope (Nikon A1R, ex/em 490/510) on a 40× objective at 37 °C in a field stimulation buffer (150 mM NaCl, 5.4 mM KCl, 10 mM Hepes, 2 mM MgCl_2_ Anhydrous, 1 mM Glucose, 2.5 mM Pyruvate, 5 mM Creatine, 5 mM Taurine, and 2 mM CaCl_2_, pH 7.4). Successive stimulations were applied with the MyoPacer Field Stimulator (IonOptix) at 0.5 and 1 Hz for 1 min each with a rest of 1 min in between (biphasic pulse, 40 V amplitude, and 0.5 ms delay). Normalization was done to the average resting fluorescence intensity (F/F0). Ca^2+^ transients were analyzed using a homemade program developed with Matlab and Statistics toolbox (version R2014B, The MathWorks Inc.), as previously described [14] to obtain different parameters including: peak amplitude, time to peak, half time and time peak to basal. For cardiomyocyte shortening, line scanning was performed along the long axis of Fluo-4 loaded cells. Using ImageJ, cell length was measured in both resting and maximally contracted states to calculate the shortening as a percentage of the resting cell length.

### 2.12 Metabolic assays

For oxygen consumption rates (OCR), an Oroboros respirometer was used at 25 °C to monitor basal and maximal respiration (by 10 μM FCCP) levels on freshly isolated cardiomyocytes (500 μg) in CCB supplemented with 2 mM pyruvate. Total ATP content was measured on freshly isolated adult cardiomyocytes by an ATP Bioluminescence Assay Kit (Roche 11,699,709,001). Mitochondrial superoxide ion levels were measured with a BD Fortessa-X20 flow cytometer, after a 10-min incubation of isolated cardiomyocytes with 2 μM MitoSOX Red (M36008) at 37 °C.

### 2.13 Hypoxia-reoxygenation

Cardiomyocytes plated on B35 dishes IBIDI plate with a glass bottom were washed twice to remove serum and nutrients with the Hypoxia-Reoxygenation Buffer (HRB: NaCl 140 mM, KCl 5 mM, MgCl_2_ 1 mM, HEPES 10 mM, CaCl_2_ 2 mM, pH 7.4). Hypoxia was then performed in 1 mL HRB for 70 min (including a stabilization period of 25 min to reach the desired level of O_2_) at 0.5 % O_2_ in a hypoxic incubator (Eppendorf Galaxy 48R). Reoxygenation was induced for 2 h at 37 °C and 19 % O_2_ by quickly, but gently replacing the hypoxic medium with 1.5 mL plating medium.

At the end of the reoxygenation, cells were detected by propidium iodide staining (PI, 1 μg/mL) with a laser scanning confocal microscope (Nikon A1R) at an excitation/emission spectrum of 488/595 nm. Percentage of cell death was determined from the mean of the number of PI-positive cardiomyocytes and the morphologically-dead cells, counted on ImageJ.

### 2.14 Principal component analysis (PCA)

PCA analysis was performed with R (version 4.3.1). The PCA was executed with dudi.pca() function form Ade4 package (version 1.7–22) with centered and scaled data on 3 components, according to the screen plot. For the graphic, the fviz_pca_biplot() function from FactoExtra package (version 1.0.7) was used.

#### 2.14.1 Statistical analyses

Since the normality test failed or the n number was too low, the Mann-Whitney non-parametric *t*-test was applied to all our datasets. Data are presented as median [interquartile range, IQR; 25 %–75 %]. Mouse experiments were done at least for *n* = 4 mice/group and *p* < 0.05 was considered statistically significant.

## 3 Results

### 3.1 Cardiac and metabolic characterization of the *ob/ob* mice

**Fig. 1:**
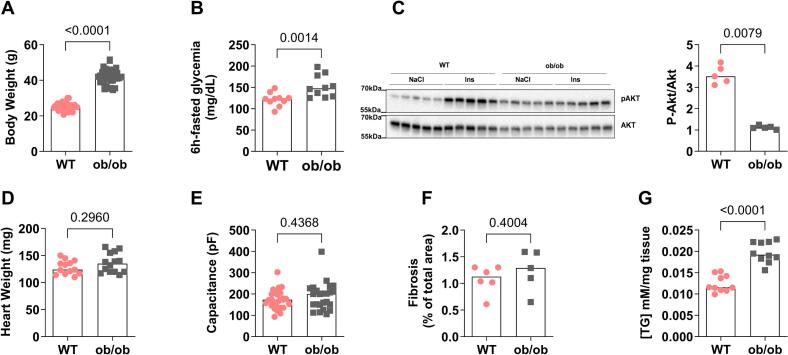
**Analysis of metabolic and cardiac phenotype of 12-week-old *ob****/****ob* mice. (A)** Body weight. *n* = 37 mice per group **(B)** Blood glucose concentration (mg/dL). *n* = 10 mice per group **(C)** Cardiac insulin sensitivity. Left panel: representative immunoblot. Right panel: quantification of p-AKT/AKT as a fold of insulin-induced AKT phosphorylation over NaCl (*n* = 5 mice per group). **(D)** Heart weight (*n* = 13 WT mice and 14 *ob/ob* mice). **(E)** Cardiomyocyte membrane capacitance recorded by patch clamp (from *n* = 27 cells isolated from 2 WT mice and *n* = 24 cells isolated from 2 *ob/ob* mice on 2 independent experimental days, respectively). **(F)** Quantification of fibrotic area of cardiac tissue after Sirius Red staining (*n* = 6 WT and 5 *ob/ob* mice). **(G)** Quantification of triglyceride concentration in cardiac tissues (n = 10 mice per group). Results are presented as median. Mann-Whitney non-parametric test was performed; *p*-value is indicated on each graph and considered significant if <0.05. (For interpretation of the references to colour in this figure legend, the reader is referred to the web version of this article.)

At the age of 12 weeks, the *ob/ob* mice displayed an increased body weight associated with hyperglycemia (Fig. 1A-B), as well as a cardiac insulin resistance reflected by decreased insulin-stimulated Ser473-AKT phosphorylation compared to WT mice (Fig. 1C**, Supplemental Fig. 1**). Echocardiography analyses revealed an anterior wall strain rate reduction with preserved fractional shortening, a decreased heart rate and no change in diastolic function (Table 1). While an increase in both anterior and posterior left ventricular wall thickness and a trend towards an increased relative wall thickness (*p* = 0.063) were observed in *ob/ob* mice (Table 1), no change was measured in either heart weight, LV mass or cardiomyocyte membrane capacitance in *ob/ob* mice compared to the WT group (Fig. 1D-E**,** Table 1), indicating a potential concentric remodeling. Sirius Red staining of collagen content showed that diabetic *ob/ob* mice did not exhibit cardiac fibrosis (Fig. 1F); but an increase in cardiac triglycerides content was observed (Fig. 1G).

**Table 1:** **Echocardiography analysis of WT and *ob/ob* mice at 12 weeks old.** Left ventricle (LV) mass, heart rate, left ventricular anterior wall diastolic and systolic thickness (AWD and AWS, respectively), left ventricular posterior wall diastolic and systolic thickness (PWD and PWS, respectively), left ventricular dimension [end diastolic diameter (LVEDD) and end systolic diameter (LVESD)], relative wall thickness (2PWD/LVEDD), fractional shortening (FS), and posterior and anterior walls strain rate (PWSR and AWSR, respectively) were measured using echocardiography in WT (n = 8) and *ob/ob* (*n* = 9) mice at 12 weeks. For diastolic function, isovolumetric relaxation time (IVRT), E/A and E/e’ ratios were assessed in WT (n = 9) and *ob/ob* (n = 14) mice at 12 weeks old. Data are presented as Median [IQR; 25 %–75 %]. Mann-Whitney non-parametric test was performed; *p*-value is indicated and considered significant if *p < 0.05.

	WT	*ob/ob*	*p-*value
*Semi-vigile (ketamine 80 mg/kg, IP)*			
LV mass, mg	83.37 [71.59,91.59]	85.65 [72.75,91.11]	0.814
Heart rate, bpm	647 [621,675]	580* [526,612]	0.023
AWD thickness, mm	0.83 [0.81,0.86]	0.82 [0.79,0.86]	0.618
AWS thickness, mm	1.40 [1.32,1.60]	1.60* [1.50,1.60]	0.027
PWD thickness, mm	0.81 [0.75,0.85]	0.87* [0.83,0.96]	0.035
PWS thickness, mm	1.40 [1.25,1.60]	1.60* [1.55,1.70]	0.007
LVEDD, mm	3.25 [3.05,3.47]	3.00 [2.90,3.40]	0.370
LVESD, mm	1.70 [1.27,1.85]	1.40 [1.25,1.55]	0.165
Relative wall thickness	0.49 [0.44,0.54]	0.58 [0.51,0.64]	0.063
LV FS, %	48.25 [44.60,54.33]	54.40 [50.90,59.70]	0.160
PWSR, unit/s	22 [20,23]	21 [16,22]	0.401
AWSR, unit/s	22.50 [21.00,24.75]	19.00* [17.50,21.5]	0.017

*Anesthetized (2–3* *% isoflurane)*			
Heart rate, bpm	400 [385,410]	453* [402,464]	0.008
IVRT, ms	14.58 [9.97,17.70]	16.34 [11.47,24.23]	0.158
E/A	1.32 [1.12,1.79]	1.25 [1.03,1.71]	0.543
E/e’	41.60 [34.75,57.40]	45.50 [38.63,56.15]	0.758

Altogether, our data demonstrate that the 12-week-old *ob/ob* mice represent a T2D obese mouse model that develops only a modest DCM with strain rate dysfunction and concentric remodeling, thereby being an interesting model to study the impact of mainly T2D and obesity on MAMs.

### 3.2 Identification and functional enrichment analysis of cardiac MAM proteins in the *ob/ob* model

**Fig. 2:**
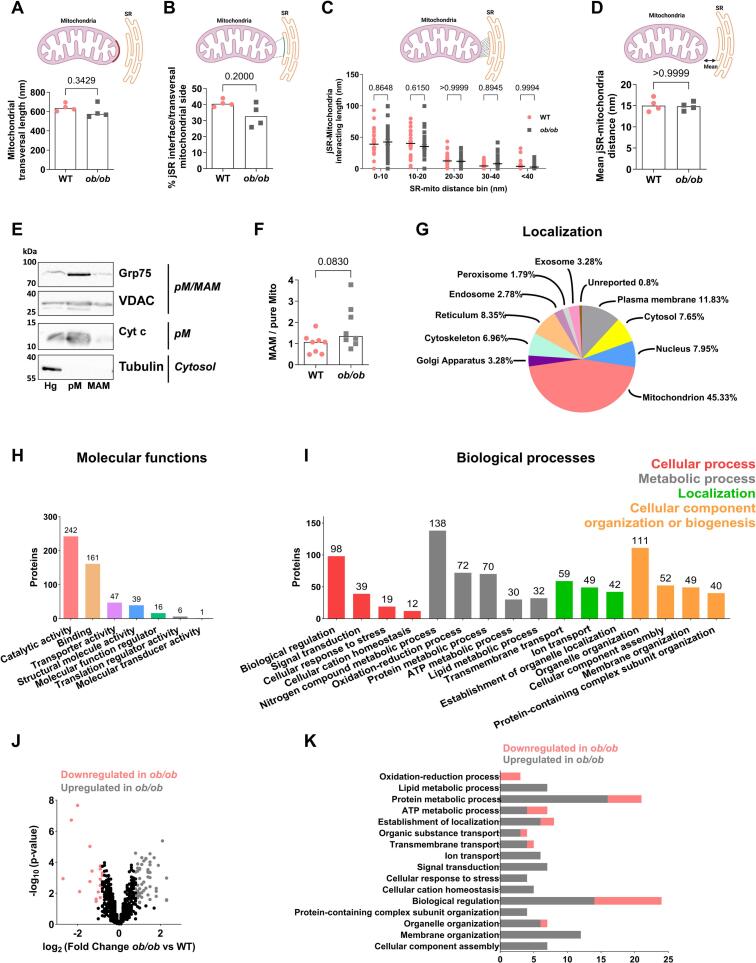
**Characterization of cardiac MAMs from WT and *ob/ob* mice.** **(A-D)** Ultrastructural analysis by electron microscopy of the cardiac junctional sarcoplasmic reticulum (jSR)–mitochondria interactions in isolated cardiomyocytes. Quantification of the length of the mitochondrial transversal side **(A)** and of the jSR–mitochondria interface **(B)** (*n* = 4 mice per group, representing the mean of 5–11 contacts per mouse). **(C)** Bar graph shows the frequency of jSR–mitochondria interaction width (*n* = 30 contacts analyzed from 4 WT mice and *n* = 35 contacts from 4 *ob/ob* mice). **(D)** Quantification of the jSR-mitochondria mean width distance (n = 4 mice per group, representing the mean of 5–11 contacts per mouse). **(E)** Representative immunoblotting of heart fractionation showing the purity of the enriched MAM fraction with MAM proteins, Grp75 and VDAC, and absence of pure mitochondrial and cytosolic proteins, Cytochrome c and tubulin. pM: pure mitochondria; MAM: mitochondria associated reticular membranes; Hg: homogenate. **(F)** Quantification of the WT-relative protein ratio of MAMs over pure mitochondria from heart fractionation at 12 weeks (*n* = 8 mice per group). **(G-K)** Process enrichment analysis of differentially expressed proteins of the cardiac MAMs (*n* = 4 mice per group). **(G)** Localization of cardiac MAM proteins in various organelles. **(H)** Classification of the identified proteins into molecular functions. **(I)** Repartition of MAM proteins in the 4 major biological processes identified and their subgroups. **(J)** Volcano plot of the 857 proteins identified in the WT and *ob/ob* MAM proteome. Each point represents an individual protein. Significant upregulated and downregulated proteins in *ob/ob* versus WT are presented in grey and pink, respectively. **(K)** Bar graph showing the functional annotation of the main processes differentially expressed in WT and *ob/ob* cardiac MAMs. Results are presented as median. Mann-Whitney non-parametric test was performed; *p*-value is indicated on each graph and considered significant if <0.05. (For interpretation of the references to colour in this figure legend, the reader is referred to the web version of this article.)

To investigate whether obesity and T2D specifically affect the cardiac MAM structure and composition in the *ob/ob* model, we first assessed the junctional sarcoplasmic reticulum (jSR) and mitochondria associations in WT and *ob/ob* cardiomyocytes by transmission electron microscopy. Similar length and width of the MAMs were observed between the two groups (Fig. 2A-D), suggesting no alterations in the thickness of the *ob/ob* cardiac MAMs. Next, we performed an enriched MAM isolation by heart fractionation: purity of the enriched MAM fractions was confirmed by immunoblotting (Fig. 2E**, Supplemental Fig. 2**), as previously validated [33]. Quantification of MAM and total mitochondrial proteins revealed a trend towards an increase in the ratio of MAM protein content to that of pure mitochondria in the *ob/ob* compared to WT hearts (Fig. 2F). Isolated cardiac MAMs were then analyzed by MS-based quantitative proteomics: 857 proteins were repeatedly identified and quantified from four biological replicates of WT and *ob/ob* MAM fractions (Supplemental Table 1). Interestingly, some well-known MAM proteins were detected in our samples, such as Calnexin, SERCA2 (Atp2a2), VDAC, BiP, VAPB, Fundc2 and Mfn1 [34]. The composition of the cardiac MAMs was analyzed through determining first the compartmental distribution of the identified proteins using the UniProt resource. As presented in Fig. 2G, most proteins were mitochondrial proteins (45.33 %), and proteins localized in the plasma membrane (11.83 %), reticulum (8.35 %) and cytoskeleton (6.96 %). Other subcellular locations encompassed various subcellular compartments, notably nucleus, Golgi and exosomes. Importantly, the repartition for the localization of the identified proteins, mainly in mitochondria, plasma membrane, reticulum and cytoskeleton, is consistent with a pronounced enrichment of MAMs, but may also derive from plasma membrane-mitochondria junctions. We next assessed the molecular functions of the identified proteins. The major protein type detected covered the proteins with catalytic activity (242 proteins) and, secondly, the binding proteins (161 proteins) (Fig. 2H). The remaining proteins were transporters, proteins with structural molecule activity or transcriptional and translational regulators. These molecular functions of the cardiac MAM-enriched proteins are consistent with the well-described hotspot role of the MAMs in the cell, notably for energy metabolism, Ca^2+^ fluxes and cell signaling. Functional annotation of MAM proteins was later performed using the Panther software, revealing four major biological processes in the cardiac MAMs: cellular process, metabolic process, localization and cellular component organization or biogenesis (Fig. 2I). Inside the cellular process, the most prominent functions were the biological regulation and the signal transduction, key features of the MAMs. Concerning the metabolic processes, the main metabolic pathways highlighted were for nitrogen compound, oxido-reduction, ATP and lipid metabolism. The biological process termed localization referred mainly to transmembrane and ion transport, while the cellular component organization relied on organelle and membrane organization (Fig. 2I). Altogether, the exhaustive characterization of the cardiac MAMs of the WT-*ob/ob* dataset supports the known roles and functions of MAMs in the cardiomyocyte.

Relative quantification of proteins in cardiac MAMs purified from WT and *ob/ob* mice revealed 61 proteins enriched in *ob/ob* MAMs whereas only 19 were enriched in WT MAM (Fig. 2J**,** Supplemental Table 3). An enrichment analysis of the whole MAM protein dataset, using the Panther software, revealed 24 upregulated biological processes and only 2 downregulated ones in the *ob/ob* mice compared to WT ones (Supplemental Table 4**).** Functional annotation of the differentially expressed MAM proteins performed using the Panther software, further supported an upregulation of most biological processes in the *ob/ob* model compared to WT, notably: the lipid metabolism including several Apoliproteins and perilipin-4, the ion transport with the Ryanodine receptor 2 and Ankyrin-1, the cellular response to stress and the membrane organization encompassing several spectrins (Fig. 2K). However, only the ATP oxidation-reduction process exhibits downregulated proteins such as Pyruvate dehydrogenase kinase 4 Pdk4 and D-beta-hydroxybutyrate dehydrogenase BDH1, while the biological regulation and the ATP metabolic processes showed both up- and down-regulation in *ob/ob* cardiac MAMs (Fig. 2K).

To conclude, our proteomic analysis of cardiac MAMs identified key functions for the heart functioning, notably the energy metabolism, thus revealing the biological relevance of the cardiac MAMs in the heart and so, in cardiovascular pathologies. A few processes seem upregulated in the *ob/ob* cardiac MAMs, deserving further experiments.

### 3.3 Functional validation of the main altered processes in the *ob/ob* model

**Fig. 3:**
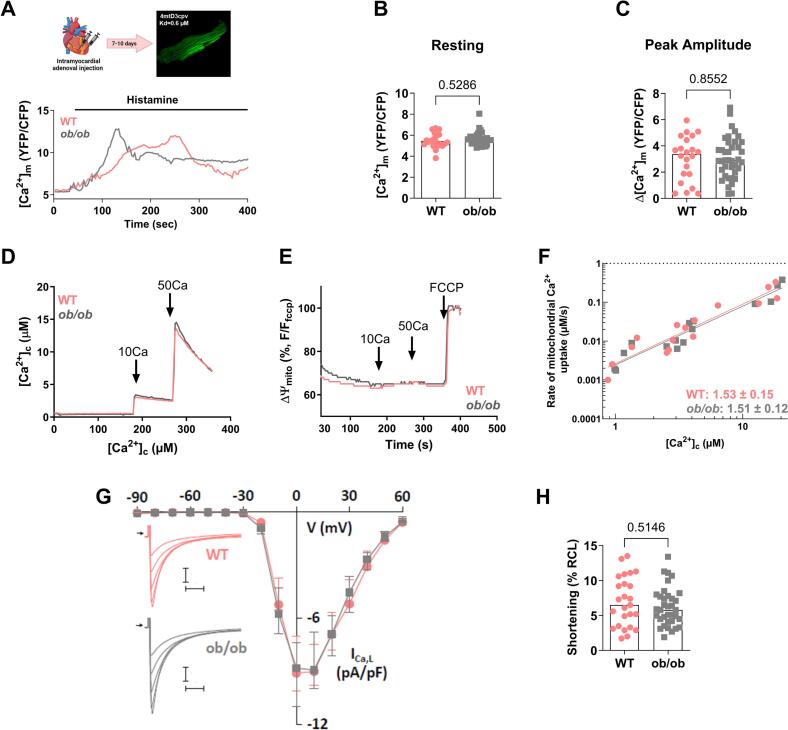
**Mitochondrial Ca**^**2+**^**dynamics and contractile function in WT and *ob/ob* cardiomyocytes. (A)** Representative traces of the [Ca^2+^]_m_ as a YFP/CFP ratio showing the histamine-driven IP3R stimulation in WT and *ob/ob* cardiomyocytes. **(B)** Quantification of the resting [Ca^2+^]_m_ fluorescence ratio (*n* = 22 cells from 4 WT mice and 39 cells from 4 *ob/ob* mice, on 4 independent experimental days). **(C)** Mitochondrial [Ca^2+^] peak amplitude after histamine-driven IP3R stimulation, as a result of the difference between the resting [Ca^2+^]_m_ and the [Ca^2+^]_m_ peak level (n = 22 cells from 4 WT mice and 39 cells from 4 *ob/ob* mice, on 4 independent experimental days). **(D)** Representative time courses of the mitochondrial clearance of the [Ca^2+^]_c_ rise, measured by Fura2, upon addition of CaCl_2_ bolus (10 μM followed by 50 μM) in WT or *ob/ob* cardiac mitochondria suspensions**. (E)** Simultaneous recordings of mitochondrial membrane potential with **(D)**, measured by TMRM and calibrated for maximal depolarization with 2 μM FCCP. **(F)** Double logarithmic plot of the initial Ca^2+^ uptake rates against the measured peak [Ca^2+^]_c_ in WT and *ob/ob* cardiac mitochondria (n = 3 mice per group on 3 independent experimental days). Slope of each linear fit is indicated. **(G)** Current-voltage relationships of normalized peak L-type Ca^2+^ current to membrane capacitance from WT and *ob/ob* cells. Data are displayed as medians with interquartile ranges (from *n* = 11 cells isolated from 2 WT and *n* = 14 cells isolated from 2 *ob/ob* mice, on 2 independent experimental days). The insert shows representative L-type Ca^2+^ current traces recorded during depolarizing steps spaced 10 mV apart and varying between 0 (lower trace) and + 40 mV (upper trace) from a holding potential of −80 mV. Horizontal bars, 50 ms. Vertical bars, 2 pA/pF. Arrows indicate zero current level. **(H)** Cardiomyocyte contractility under 1 Hz field stimulation, presented as a percentage of the resting cell length (RCL) (*n* = 25 cells from 4 WT mice and 35 cells from 4 *ob/ob* mice on 4 independent experimental days). Results are presented as median. Mann-Whitney non-parametric test was performed; *p*-value is indicated on each graph and considered significant if <0.05.

We then investigated whether the observed changes in the major biological MAM processes of the *ob/ob* mice will translate into functional cardiomyocyte alterations. In our previous study, we reported that the cardiac MAMs from the HFHSD mice displayed a reduction in the ATP metabolism, protein and ion transport processes, leading to reduced SR-mitochondrial Ca^2+^ transfer and bioenergetics [14]. Since we observed an enrichment of proteins involved in the ion transport and cellular cationic homeostasis processes in *ob/ob* vs WT cardiac MAMs, we first analyzed the SR-mitochondrial Ca^2+^ transfer through IP3R in isolated cardiomyocytes by measuring the [Ca^2+^]_m_ using an adenoviral injection of the FRET-based sensor, 4mtD3cpv (Fig. 3A**)**. No significant changes in the resting [Ca^2+^]_m_ and in the histamine-induced mitochondrial Ca^2+^ peak amplitude were observed between WT and *ob/ob* cardiomyocytes (Fig. 3B-C). Next, we investigated whether the mitochondrial Ca^2+^ uptake, through the mitochondrial Ca^2+^ uniporter, would be affected in *ob/ob* cardiac cells. Isolated heart mitochondria were exposed to micromolar [Ca^2+^] and a rapid mitochondrial Ca^2+^ clearance was measured in both WT and *ob/ob* groups (Fig. 3D). Simultaneously, the measurement of mitochondrial membrane potential showed similar mitochondrial polarization, reflecting that the mitochondrial driving force was not a limiting factor for the mitochondrial Ca^2+^ uptake (Fig. 3E). Double logarithmic plots of the initial Ca^2+^ uptake rates against cytosolic [Ca^2+^] showed coincident linear fit lines with similar slopes, highlighting no change in either threshold or cooperative activation of the uniporter (Fig. 3F). Thus, the function of the mitochondrial Ca^2+^ uniporter was not affected between WT and *ob/ob* hearts, indicating that the MAM-driven Ca^2+^ coupling remains unchanged.

To determine if the excitation-contraction coupling was altered in *ob/ob* mice, we performed electrophysiological recordings of the inward L-type Ca^2+^ current (ICa,L) in WT and *ob/ob* ventricular myocytes: similar density of ICa,L was observed between both groups (Fig. 3G). We then assessed the cytosolic Ca^2+^ transients by Fluo4-AM in cardiomyocytes under field stimulation at both 0.5 and 1 Hz. Our results revealed a similar peak amplitude between both groups, an increased time to peak, while the half time and the time peak to basal were significantly decreased at 0.5 Hz in the *ob/ob* cardiomyocytes compared to WT mice, and only the half time was reduced at 1 Hz in the *ob/ob* cells (Table 2). Assessment of cardiomyocyte shortening under field stimulation, reflecting cardiomyocyte contractility, did not reveal a significant difference between WT and *ob/ob* groups (Fig. 3H).

**Table 2:** **Ca**^**2+**^**transient characteristics in WT and *ob/ob* cardiomyocytes, under 0.5 Hz and 1** **Hz field stimulation.** Data are displayed as median [IQR; 25 %, 75 %] from n = numbers of cells indicated in the table, from 4 mice per group, on 4 independent experimental days. F/F_0_: peak amplitude; TTP: time to peak; TPB: time peak to basal; t_1/2_: half-time. Mann-Whitney non-parametric test was performed; *p*-value is indicated and considered significant if *p < 0.05.

Field stimulation frequency	0.5 Hz			1 Hz		
Group	WT	*ob/ob*	*p-*value	WT	ob/ob	*p-*value
n (cells)	98	102		96	94	
F/F_0_	1.09[0.64,1.55]	1.07[0.64,1.61]	0.894	0.82[0.50,1.03]	0.91[0.55,1.41]	0.077
TTP (s)	0.22[0.16,0.39]	0.32*[0.19,0.50]	0.009	0.12[0.09,0.18]	0.13[0.11,0.22]	0.073
TPB (s)	1.74[1.55,1.81]	1.62*[1.44,1.76]	0.007	0.86[0.82,0.88]	0.84[0.77,0.87]	0.894
t_1/2_ (s)	0.32[0.21,0.43]	0.25*[0.18,0.35]	0.001	0.24[0.19,0.32]	0.19*[0.16,0.25]	<0.0001

**Fig. 4:**
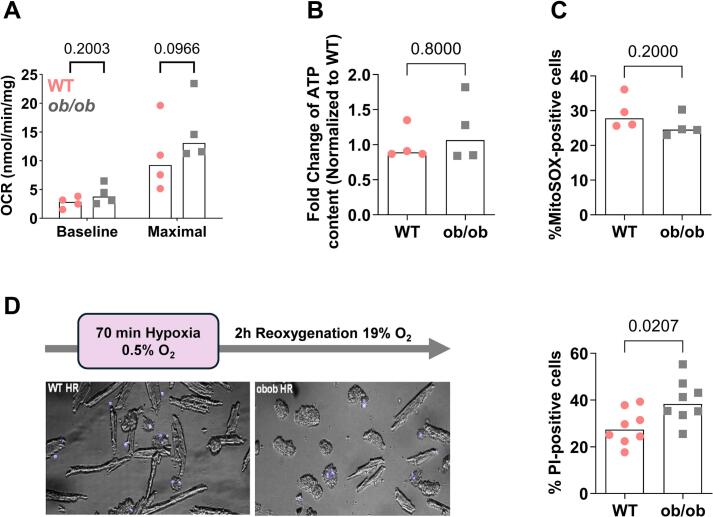
**Metabolic and viability assessments of *ob/ob* and WT mouse cardiomyocytes. (A)** OCR measurement by oxygraphy: baseline and FCCP-induced maximal oxidative phosphorylation was measured in intact cardiomyocytes (*n* = 4 mice per group on 4 independent experimental days). **(B)** Fold change of total ATP content in freshly isolated WT and *ob/ob* cardiomyocytes. *Ob/ob* values were normalized to the respective WT values (n = 4 mice per group). **(C)** ROS production measured by flow cytometry using MitoSOX assay in freshly isolated WT and *ob/ob* cardiomyocytes (n = 4 mice per group on 4 independent experimental days). **(D)** WT and *ob/ob* cardiomyocytes were subjected to 70 min hypoxia followed by 2 h reoxygenation. Representative images (left panel) and quantitative analyses (right panel) of cardiomyocyte cell death after hypoxia/reoxygenation determined by propidium iodide-positive cells (*n* = 8 mice per group, on 8 independent experimental days). Results are presented as median. Mann-Whitney non-parametric test was performed; *p*-value is indicated on each graph and considered significant if <0.05.

Finally, we investigated mitochondrial energetic processes. Oxygen consumption rates (OCR) were measured on intact cardiomyocytes and no changes in either basal or FCCP-induced maximal respiration were observed between both groups (Fig. 4A). Neither the total ATP content was affected in the *ob/ob* cardiomyocytes (Fig. 4B), nor the mitochondrial ROS content (Fig. 4C). To investigate the potential effect of the upregulated “cellular response to stress” process seen in the *ob/ob* cardiac MAM proteome, we evaluated cell death after a hypoxia/reoxygenation stress on isolated cardiomyocytes, as a stress-induced mitochondrial dysfunction experiment: cell death was increased in *ob/ob* cardiomyocytes under hypoxic stress (Fig. 4D), while being similar in normoxic conditions (WT: 29 % [27,34] vs *ob/ob*: 28 % [25,29], p = ns). Immunoblotting of ER stress markers, Grp78, phospho-eIF2α and CHOP, and autophagy signaling, phopsho-AMPK and LC3, did not reveal any differences between WT and *ob/ob* hearts (**Supplemental Fig. 3).**

### 3.4 Multivariate analysis to compare with the high-fat high-sucrose diet model of DCM

**Fig. 5:**
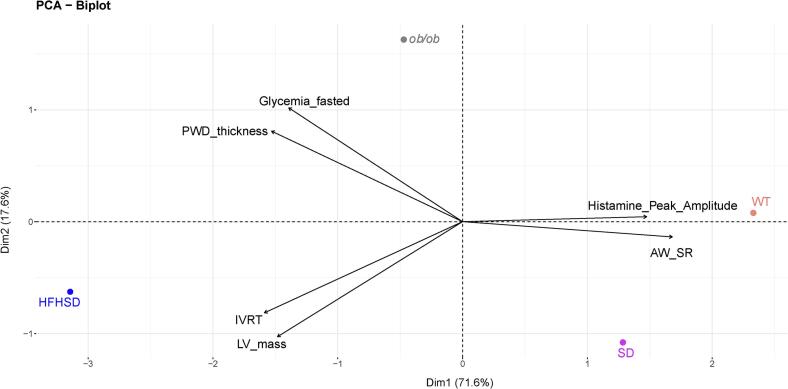
**Principal component analysis (PCA) shown with a biplot of groups of interests and accounting variables.** The multi-variate scattering is displayed by the first and second principal components. The four groups are represented by points: standard diet (SD, purple), and high-fat high-sucrose diet (HFHSD, blue), wild type (WT, orange) and *ob/ob* (grey). Accounting variables participating in PCA are represented by arrows. (For interpretation of the references to colour in this figure legend, the reader is referred to the web version of this article.)

We took advantage of our previous dataset, with similar experiments as in this study, that was performed on the HFHSD mouse model of DCM with early HFpEF symptoms [14,35], to realize a principal component analysis (PCA) with the dataset of this study (**Supplemental Table 5, Supplemental Fig. 4**). PCA demonstrated a divergence between the HFHSD group and the three others when considering all the variables from the dataset. Focusing on the main contributing variables (**Supplemental Table 6)**, the HFHSD group still diverged from the other groups, as displayed by their scattering along the first PC (71.6 % of variance), contributed mainly by a decreased strain rate (AW SR) and a reduced histamine-induced Ca^2+^ peak amplitude (Fig. 5). Along the second dimension, HFHSD and *ob/ob* further diverged with higher glycemia and wall thickness for the *ob/ob*, and increased IVRT and LV mass for the HFHSD.

## 4 Discussion

In this paper, we reported an exhaustive MS-based proteomic characterization of the cardiac MAM proteome for the first time. Furthermore, we analyzed the impact of obesity and T2D on cardiac MAMs using a genetic mouse model of combined obesity and T2D, with only early symptoms of DCM, i.e. LV concentric remodeling and subtle strain rate dysfunction. While we observed no change in the cardiac MAM structure in the *ob/ob* heart, we revealed an enrichment of proteins involved in most biological processes of the cardiac MAM proteome, notably the lipid and protein metabolism, the ion transport, the cellular response to stress and the membrane organization; however, without clear translation into functional changes. Interestingly, we previously studied a diet-induced obesogenic T2D mouse model of DCM with early cardiometabolic HFpEF where we identified the SR-mitochondria Ca^2+^ uncoupling as a trigger of the cardiac dysfunction [14]. Comparison of these two datasets suggests that the main upregulated processes in the *ob/ob* cardiac MAMs may be associated with their strong hyperglycemia and obesity phenotype, and their subsequent lipotoxicity; while the SR-mitochondria Ca^2+^ uncoupling observed in the HFHSD mice may be rather connected to the diastolic dysfunction and concentric hypertrophy, key hallmarks of cardiometabolic HFpEF.

To date, proteomic analyses of MAMs have been reported mainly in mouse brain and liver [[16], [17], [18], [19]]. Comparing our study with previous ones, we reported that the compartmental distribution of the identified cardiac MAM proteins is pretty similar to the liver and brain ones, especially for reticular and cytoskeleton proteins, except that cardiac MAMs display an enrichment of mitochondrial proteins towards less plasma membrane proteins compared to brain [16]. This change in proportion could be explained by the higher number of mitochondria in cardiomyocytes. Importantly, all MAM proteomic analyses from tissues report the presence of proteins from other compartments than mitochondria and reticulum, such as cytoskeleton and plasma membrane. One could speculate that these proteins refer to the cytoskeleton regulation of the MAMs, to the plasma membrane-mitochondria interactions with a potential interplay with MAMs, as recently discovered for the MAMs and lipid droplets [36]. Nevertheless, we cannot exclude that these proteins represent unspecific proteins outside of MAMs. At the level of molecular functions, cardiac MAMs present mainly enzymes and transporters as previously observed in brain MAMs [17]. These similarities were also confirmed at the level of the major biological processes encountered in cardiac MAMs, i.e. cellular and metabolic processes, localization and cellular component organization or biogenesis, which were previously reported in brain and liver MAMs [16,17]. Therefore, our exhaustive MS-based proteomic characterization of the cardiac MAMs supports that MAM functions are well conserved across tissues, highlighting the crucial involvement of MAMs in many vital processes, regardless of the organ.

In the *ob/ob* heart, our MAM proteomic analysis suggests a MAM rearrangement towards upregulated processes, mainly in lipid metabolism, response to stress and ion transport. While we did not observe any enhanced functional SR-mitochondria Ca^2+^ coupling or ER stress in the *ob/ob* cardiomyocyte, these cells were more prone to cell death after a hypoxic stress. Interestingly, we reported the increased protein expression of Apolipoprotein E, ApoE, in *ob/ob* MAMs. The mitochondrial localization of ApoE has recently emerged with a potential role in MAMs, via a direct interaction with the IP3R-VDAC Ca^2+^ channeling complex [37]. Notably, ApoE involvement in MAMs has been linked to the development of neurodegenerative diseases, such as Alzheimer's disease [38], in which mitochondrial Ca^2+^ overload and increased SR-mitochondria Ca^2+^ coupling are key triggers of the cell death-induced neurodegeneration. Thus, together with its known role in lipid metabolism, the ApoE-triggered SR-mitochondria Ca^2+^ coupling may also contribute to the increased response to acute stress observed in the *ob/ob* cardiomyocyte, such as hypoxia-reoxygenation.

Regarding mitochondrial metabolism, our MS analysis revealed both up- and downregulated MAMs proteins of the respiratory chain in *ob/ob* hearts, while the mitochondrial respiration was so far preserved with even a trend towards an increased maximal respiration in *ob/ob* cardiomyocytes. Similar findings were observed by cardiac proteomic analysis in another genetic model of DCM, the *db/db* mice [39]. This may suggest an attempt of the diabetic heart to augment mitochondrial ATP production in the early stage of the pathology, as also observed in human myocardium with differences between metabolically healthy obese and T2D patients [40]. On a therapeutic side, the cardiolipin-stabilizing drug elamipretide was recently shown to improve mitochondrial respiration in myocardium of HF [41] or hypertrophic cardiomyopathy [42] patients. Since cardiolipin is an important mitochondrial lipid, notably involved in the regulation of the mtCU [43] and IP3R [44], further research is needed to determine if elamipretide may improve the MAM organization in DCM.

Additionally, we took advantage of our *ob/ob* model to assess the specific impact of both obesity and T2D on the cardiac MAM composition and function, in order to compare with our previous study in a diet-induced model of DCM [14]. While the differentially expressed major biological processes were pretty similar between the HFHSD and the *ob/ob* models, the alterations towards up- or down-regulation were clearly different. Only the cellular response to stress and the lipid metabolism processes were reported to be upregulated in both models, which could rather rely on their common obese phenotype and the subsequent lipotoxicity. As for the processes relevant to cardiac contractile function, Ca^2+^ transport and ATP metabolism were differentially altered in HFHSD and *ob/ob* cardiac MAMs. The HFHSD cardiomyocytes display a reduction of the MAM-driven Ca^2+^ fluxes, leading to reduced mitochondrial Ca^2+^ content and energetics [14]. In the *ob/ob* cardiomyocyte, while these processes were upregulated, we here demonstrated so far no alteration of the SR-mitochondria Ca^2+^ fluxes, of the mitochondrial Ca^2+^ uniporter and of mitochondrial bioenergetics at the age of 12 weeks old. Whether an increased SR-mitochondria Ca^2+^ coupling occurs with time in the *ob/ob* cardiomyocyte remains to be investigated, as well as if it could be related to their preponderant hyperglycemia, as previously reported in the mouse neonatal cardiomyocytes exposed to high glucose [13]. One explanation of the different outcomes in MAMs between the *ob/ob* and HFHSD mice may be the different origin of their obesity and T2D: genetic versus diet-induced.

Importantly, it must be noted that even if T2D and obesity alter some biological processes in the cardiac MAMs of *ob/ob* mice, the fold change and the number of the differentially expressed proteins are rather small: this suggests that the MAM protein composition is rather stable in the heart despite T2D and obesity. MAMs are known to be very dynamic structures, but the complex ultrastructure of the cardiomyocyte may limit their dynamism. Thus, the altered MAM function in T2D hearts may not only depend on its protein composition but also on the length and width of the SR-mitochondria interactions. Effectively, we previously demonstrated by electron microscopy analysis that the MAM interface in the HFHSD cardiomyocyte presents the same length as the SD cells, but with increased portions at a narrower width (<10 nm) [14], supporting rather the lipid metabolism than Ca^2+^ transport [45].

Our study presents some limitations. The use of this mouse model could not allow to decipher the contribution between T2D and obesity. One should remain aware that the MAM isolation is an enrichment of the SR-mitochondria interactions fraction, which could potentially lead to misleading analyses. However, our compartmental and functional analyses are 1) in line with previously reported data on brain and liver [16,17], and 2) display a strong enrichment of mitochondrial and reticular proteins, known MAM proteins and functions.

## 5 Conclusions

Our present study, in comparison with our previous one [14], complements our knowledge on the role of MAMs, especially of the SR-mitochondria Ca^2+^ coupling, during the development of T2D and obesity-related cardiac dysfunction. On one hand, increased cardiac MAM processes, as a consequence of obesity and T2D, potentially evolve towards increased SR-mitochondria Ca^2+^ coupling and cell death [13], and induce DCM with systolic dysfunction (reduced ejection fraction). On the other hand, a reduction of the SR-mitochondria Ca^2+^ coupling and of the ensuing bioenergetics, may rather trigger diastolic dysfunction and cardiac hypertrophy, key features of DCM evolving towards cardiometabolic HFpEF.

The following are the supplementary data related to this article.

Supplemental Table 1 The ARRIVE guidelines 2.0: author checklist. Supplemental Table 1

Supplemental Table 3 MS-based label-free quantitative proteomic analysis of MAMs purified from WT and *ob/ob* (OB) mice hearts (4 biological replicates per condition). Supplemental Table 3

Supplemental Table 4 Panther enrichment test (Bonferroni). Supplemental Table 4

Supplementary material Image 1

## CRediT authorship contribution statement

**Hala Guedouari:** Writing – original draft, Investigation, Formal analysis, Data curation. **Maya Dia:** Writing – original draft, Methodology, Investigation, Formal analysis, Data curation, Conceptualization. **Juliette Geoffray:** Writing – review & editing, Investigation, Formal analysis. **Camille Brun:** Writing – review & editing, Investigation, Formal analysis. **Florentin Moulin:** Investigation. **Lucas Givre:** Writing – review & editing, Investigation, Formal analysis. **Lucid Belmudes:** Investigation, Formal analysis. **Christelle Leon:** Investigation. **Stephanie Chanon:** Investigation, Formal analysis. **Jingwei Ji-Cao:** Methodology. **Christophe Chouabe:** Writing – review & editing, Investigation, Formal analysis. **Sylvie Ducreux:** Investigation, Formal analysis. **Claire Crola Da Silva:** Investigation, Formal analysis. **Ludovic Gomez:** Methodology, Investigation. **Yohann Couté:** Writing – review & editing, Investigation, Funding acquisition, Formal analysis. **Helene Thibault:** Writing – review & editing, Investigation, Formal analysis. **Jennifer Rieusset:** Writing – review & editing, Investigation, Funding acquisition, Conceptualization. **Melanie Paillard:** Writing – review & editing, Writing – original draft, Visualization, Validation, Supervision, Project administration, Methodology, Investigation, Funding acquisition, Formal analysis, Data curation, Conceptualization.

## Ethics approval and consent to participate

All animal experiments have been approved by the French Ministry of Research (#15627–2018062118508398; LYONSUD-2012-002).

## Funding

The study was founded by grants from 10.13039/501100004431Fondation de France (n°00056853 & n° 00107048) to MP and JR, from the 10.13039/501100001665Agence Nationale de la Recherche (ANR- 20-CE14-0013-01) to MP, and by a contribution from the 10.13039/100010796Azm and Saade association. MP was a recipient of postdoctoral fellowships from the Fondation Lefoulon-Delalande and Fondation pour la Recherche Médicale (n°ARF20160936149). HG was a recipient of a postdoctoral fellowship from the Fondation pour la Recherche Médicale (SPF202110014186). The authors would like to acknowledge the National Council for Scientific Research of Lebanon (CNRS-L), 10.13039/501100002708Agence Universitaire de la Francophonie (AUF) and 10.13039/501100004113Lebanese University for granting a doctoral fellowship to MD (AUF-CNRS-UL program). The proteomic experiments were partially supported by Agence Nationale de la Recherche under projects ProFI (Proteomics French Infrastructure, ANR-10-INBS-08) and GRAL, a program from the Chemistry Biology Health (CBH) Graduate School of University Grenoble Alpes (ANR-17-EURE-0003).

## Declaration of competing interest

The authors declare that they have no competing interests.

## Data Availability

The dataset generated and analyzed during the current study is available via the PRIDE partner repository with the dataset identifier PXD054672 (https://www.ebi.ac.uk/pride/login). Other data will be made available by contacting the corresponding authors, upon reasonable request.

## References

[1] Dunlay S.M., Roger V.L., Redfield M.M. (2017). Epidemiology of heart failure with preserved ejection fraction. Nat Rev Cardiol.

[2] Saeedi P., Petersohn I., Salpea P., Malanda B., Karuranga S., Unwin N. (2019). Global and regional diabetes prevalence estimates for 2019 and projections for 2030 and 2045: results from the international diabetes federation diabetes atlas, 9(th) edition. Diabetes Res Clin Pract.

[3] Bugger H., Abel E.D. (2009). Rodent models of diabetic cardiomyopathy. Dis Model Mech.

[4] Chen Y., Hua Y., Li X., Arslan I.M., Zhang W., Meng G. (2020). Distinct types of cell death and the implication in diabetic cardiomyopathy. Front Pharmacol.

[5] Dillmann W.H. (2019). Diabetic cardiomyopathy. Circ Res.

[6] de Simone G., Devereux R.B., Chinali M., Lee E.T., Galloway J.M., Barac A. (2010). Diabetes and incident heart failure in hypertensive and normotensive participants of the strong heart study. J Hypertens.

[7] Kannel W.B., Hjortland M., Castelli W.P. (1974). Role of diabetes in congestive heart failure: the Framingham study. Am J Cardiol.

[8] van Ham W.B., Kessler E.L., Oerlemans M., Handoko M.L., Sluijter J.P.G., van Veen T.A.B. (2022). Clinical phenotypes of heart failure with preserved ejection fraction to select preclinical animal models. JACC Basic Transl Sci.

[9] Anker S.D., Butler J., Filippatos G., Ferreira J.P., Bocchi E., Bohm M. (2021). Empagliflozin in heart failure with a preserved ejection fraction. N Engl J Med.

[10] Kosiborod M.N., Abildstrom S.Z., Borlaug B.A., Butler J., Rasmussen S., Davies M. (2023). Semaglutide in patients with heart failure with preserved ejection fraction and obesity. N Engl J Med.

[11] Lopez-Crisosto C., Bravo-Sagua R., Rodriguez-Pena M., Mera C., Castro P.F., Quest A.F. (2015). ER-to-mitochondria miscommunication and metabolic diseases. Biochim Biophys Acta.

[12] Lu B., Chen X., Ma Y., Gui M., Yao L., Li J. (2024). So close, yet so far away: the relationship between MAM and cardiac disease. Front Cardiovasc Med.

[13] Wu S., Lu Q., Ding Y., Wu Y., Qiu Y., Wang P. (2019). Hyperglycemia-driven inhibition of AMP-activated protein kinase alpha2 induces diabetic cardiomyopathy by promoting mitochondria-associated endoplasmic reticulum membranes in vivo. Circulation.

[14] Dia M., Gomez L., Thibault H., Tessier N., Leon C., Chouabe C. (2020). Reduced reticulum-mitochondria ca(2+) transfer is an early and reversible trigger of mitochondrial dysfunctions in diabetic cardiomyopathy. Basic Res Cardiol.

[15] Cherpaz M., Meugnier E., Seillier G., Pozzi M., Pierrard R., Leboube S. (2024). Myocardial transcriptomic analysis of diabetic patients with aortic stenosis: key role for mitochondrial calcium signaling. Cardiovasc Diabetol.

[16] Ma J.H., Shen S., Wang J.J., He Z., Poon A., Li J. (2017). Comparative proteomic analysis of the mitochondria-associated ER membrane (MAM) in a long-term type 2 diabetic rodent model. Sci Rep.

[17] Poston C.N., Krishnan S.C., Bazemore-Walker C.R. (2013). In-depth proteomic analysis of mammalian mitochondria-associated membranes (MAM). J Proteome.

[18] Volgyi K., Badics K., Sialana F.J., Gulyassy P., Udvari E.B., Kis V. (2018). Early Presymptomatic changes in the proteome of mitochondria-associated membrane in the APP/PS1 mouse model of Alzheimer’s disease. Mol Neurobiol.

[19] Sala-Vila A., Navarro-Lerida I., Sanchez-Alvarez M., Bosch M., Calvo C., Lopez J.A. (2016). Interplay between hepatic mitochondria-associated membranes, lipid metabolism and caveolin-1 in mice. Sci Rep.

[20] Horner S.M., Wilkins C., Badil S., Iskarpatyoti J., Gale M. (2015). Proteomic analysis of mitochondrial-associated ER membranes (MAM) during RNA virus infection reveals dynamic changes in protein and organelle trafficking. PLoS One.

[21] Zhang A., Williamson C.D., Wong D.S., Bullough M.D., Brown K.J., Hathout Y. (2011). Quantitative proteomic analyses of human cytomegalovirus-induced restructuring of endoplasmic reticulum-mitochondrial contacts at late times of infection. Molecular & cellular proteomics: MCP.

[22] Adingupu D.D., Gopel S.O., Gronros J., Behrendt M., Sotak M., Miliotis T. (2019). SGLT2 inhibition with empagliflozin improves coronary microvascular function and cardiac contractility in prediabetic Ob/Ob(−/−) mice. Cardiovasc Diabetol.

[23] Marchand A., Atassi F., Mougenot N., Clergue M., Codoni V., Berthuin J. (2016). miR-322 regulates insulin signaling pathway and protects against metabolic syndrome-induced cardiac dysfunction in mice. Biochim Biophys Acta.

[24] Percie du Sert N., Hurst V., Ahluwalia A., Alam S., Avey M.T., Baker M. (2020). The ARRIVE guidelines 2.0: updated guidelines for reporting animal research. PLoS Biol.

[25] Bartok A., Weaver D., Golenar T., Nichtova Z., Katona M., Bansaghi S. (2019). IP3 receptor isoforms differently regulate ER-mitochondrial contacts and local calcium transfer. Nat Commun.

[26] Wieckowski M.R., Giorgi C., Lebiedzinska M., Duszynski J., Pinton P. (2009). Isolation of mitochondria-associated membranes and mitochondria from animal tissues and cells. Nat Protoc.

[27] Casabona M.G., Vandenbrouck Y., Attree I., Coute Y. (2013). Proteomic characterization of Pseudomonas aeruginosa PAO1 inner membrane. Proteomics.

[28] Bouyssie D., Hesse A.M., Mouton-Barbosa E., Rompais M., Macron C., Carapito C. (2020). Proline: an efficient and user-friendly software suite for large-scale proteomics. Bioinformatics.

[29] Perez-Riverol Y., Csordas A., Bai J., Bernal-Llinares M., Hewapathirana S., Kundu D.J. (2019). The PRIDE database and related tools and resources in 2019: improving support for quantification data. Nucleic Acids Res.

[30] Wieczorek S., Combes F., Lazar C., Giai Gianetto Q., Gatto L., Dorffer A. (2017). DAPAR & ProStaR: software to perform statistical analyses in quantitative discovery proteomics. Bioinformatics.

[31] O’Connell T.D., Rodrigo M.C., Simpson P.C. (2007). Isolation and culture of adult mouse cardiac myocytes. Methods Mol Biol.

[32] Chouabe C., Espinosa L., Megas P., Chakir A., Rougier O., Freminet A. (1997). Reduction of I(Ca,L) and I(to1) density in hypertrophied right ventricular cells by simulated high altitude in adult rats. J Mol Cell Cardiol.

[33] Paillard M., Tubbs E., Thiebaut P.A., Gomez L., Fauconnier J., Da Silva C.C. (2013). Depressing mitochondria-reticulum interactions protects cardiomyocytes from lethal hypoxia-reoxygenation injury. Circulation.

[34] Silva-Palacios A., Zazueta C., Pedraza-Chaverri J. (2020). ER membranes associated with mitochondria: possible therapeutic targets in heart-associated diseases. Pharmacol Res.

[35] Dia M., Leon C., Chanon S., Bendridi N., Gomez L., Rieusset J. (2022). Effect of metformin on T2D-induced MAM ca(2+) uncoupling and contractile dysfunction in an early mouse model of diabetic HFpEF. Int J Mol Sci.

[36] Monteiro-Cardoso V.F., Giordano F. (2024). Emerging functions of the mitochondria-ER-lipid droplet three-way junction in coordinating lipid transfer, metabolism, and storage in cells. FEBS Lett.

[37] Rueter J., Rimbach G., Huebbe P. (2022). Functional diversity of apolipoprotein E: from subcellular localization to mitochondrial function. Cell Mol Life Sci.

[38] Mahley R.W. (2023). Apolipoprotein E4 targets mitochondria and the mitochondria-associated membrane complex in neuropathology, including Alzheimer's disease. Curr Opin Neurobiol.

[39] Gomes K.P., Jadli A.S., de Almeida L.G.N., Ballasy N.N., Edalat P., Shandilya R. (2022). Proteomic analysis suggests altered mitochondrial metabolic profile associated with diabetic cardiomyopathy. Front Cardiovasc Med.

[40] Montaigne D., Marechal X., Coisne A., Debry N., Modine T., Fayad G. (2014). Myocardial contractile dysfunction is associated with impaired mitochondrial function and dynamics in type 2 diabetic but not in obese patients. Circulation.

[41] Chatfield K.C., Sparagna G.C., Chau S., Phillips E.K., Ambardekar A.V., Aftab M. (2019). Elamipretide improves mitochondrial function in the failing human heart. JACC Basic Transl Sci.

[42] Nollet E.E., Duursma I., Rozenbaum A., Eggelbusch M., Wust R.C.I., Schoonvelde S.A.C. (2023). Mitochondrial dysfunction in human hypertrophic cardiomyopathy is linked to cardiomyocyte architecture disruption and corrected by improving NADH-driven mitochondrial respiration. Eur Heart J.

[43] Kamer K.J., Grabarek Z., Mootha V.K. (2017). High-affinity cooperative ca(2+) binding by MICU1-MICU2 serves as an on-off switch for the uniporter. EMBO Rep.

[44] Cannon B., Hermansson M., Gyorke S., Somerharju P., Virtanen J.A., Cheng K.H. (2003). Regulation of calcium channel activity by lipid domain formation in planar lipid bilayers. Biophys J.

[45] Giacomello M., Pellegrini L. (2016). The coming of age of the mitochondria-ER contact: a matter of thickness. Cell Death Differ.

