# Supplementary material for: Structural and functional characterization of the cardiac mitochondria-associated reticular membranes in the ob/ob mouse model
Source: J Mol Cell Cardiol Plus. 2025 May 10;12:100453. doi: 10.1016/j.jmccpl.2025.100453 (PMC12148419; doi:10.1016/j.jmccpl.2025.100453)
Supplement: Supplementary file 4 — Supplementary material [file mmc4.docx]

**SUPPLEMENTAL MATERIAL**

**Structural and functional characterization of the cardiac mitochondria-associated reticular membranes in the ob/ob mouse model**

Hala Guedouari^1*^, Maya Dia^1, 2*^, Juliette Geoffray^1^, Camille Brun^1^, Florentin Moulin^1^, Lucas Givre^1^, Lucid Belmudes^3^, Christelle Leon^1^, Stephanie Chanon^4^, Jingwei Ji-Cao^4^, Christophe Chouabe^1^, Sylvie Ducreux^1^, Claire Crola Da Silva^1^, Ludovic Gomez^1^, Yohann Couté^3^, Helene Thibault^1, 5^, Jennifer Rieusset^4†^ & Melanie Paillard^1†^

^1^ University Claude Bernard Lyon1, CarMeN Laboratory- IRIS Team, INSERM, INRA, 69500 Bron, France

^2^ Laboratory of Experimental and Clinical Pharmacology, Faculty of Sciences, Lebanese University-Beirut, Lebanon

^3^ Univ. Grenoble Alpes, INSERM, CEA, UA13 BGE, CNRS, CEA, FR2048, 38000 Grenoble, France

^4^ University Claude Bernard Lyon1, CarMeN Laboratory– MERISM team, INSERM, INRA, 69921 Oullins, France

^5^ Hospices Civils de Lyon, 69500 Bron, France

* These authors share the first authorship

^†^ These authors share the last authorship

**Correspondence:**

Dr. Melanie Paillard

U1060 CARMEN, Equipe 5- Cardioprotection

Groupement Hospitalier Est, Bâtiment B13

59 boulevard Pinel

69500 BRON, FRANCE

Tel: +33 (0)4.78.78.56.10

E-mail : [melanie.paillard@univ-lyon1.fr](mailto:melanie.paillard@univ-lyon1.fr)

Twitter: @PaillardMel

Page 3: Supplemental table 1: The ARRIVE guidelines 2.0: author checklist (see pdf attached)

Page 4: Supplemental Table 2: Gene Ontology terms corresponding to biological process, extracted from Panther software.

Page 5: Supplemental Table 3: MS-based label-free quantitative proteomic analysis of MAM purified from WT and ob/ob (OB) mouse hearts (4 biological replicates per condition) (see Excel attached)

Page 6-7: Supplemental Table 4: Dataset used to perform the principal component analysis (see Excel attached)

Page 8: Supplemental Table 5: Contribution of each variable to the dimensions of biplot from Supplemental Figure 1.

Page 9: Supplemental Figure 1: Uncropped membranes of Figure 1C

Page 10: Supplemental Figure 2: Uncropped membranes of Figure 2E

Page 11: Supplemental Figure 3: Biplot of the 4 groups with all the variables

**Supplemental table 1: The ARRIVE guidelines 2.0: author checklist (see pdf attached)**

**Supplemental Table 2: Gene Ontology terms corresponding to biological process, extracted from Panther software.**

| Biological process | Gene Ontology Terms |
| --- | --- |
| Cellular process | **GO:0009987** |
| Biological regulation | GO:0065007 |
| Cellular response to stress | GO:0033554 |
| Cellular cation homeostasis | GO:0030003 |
| Signal transduction | GO:0007165 |
| Metabolic process | **GO:0008152** |
| Lipid metabolic process | GO:0006629 |
| Nitrogen compound metabolic process | GO:0006807 |
| Protein metabolic process | GO:0019538 |
| ATP metabolic process | GO:0046034 |
| Oxidation-reduction process | GO:0055114 |
| Localization | **GO:0051179** |
| Ion transport | GO:0006811 |
| Transmembrane transport | GO:0055085 |
| Intracellular protein transport | GO:0006886 |
| Establishment of organelle localization | GO:0051656 |
| Cellular component organization or biogenesis | **GO:0071840** |
| Organelle organization | GO:0006996 |
| Cellular component assembly | GO:0022607 |
| Membrane organization | GO:0061024 |
| Protein-containing complex subunit organization | GO:0043933 |

**Supplemental table 3: MS-based label-free quantitative proteomic analysis of MAM purified from WT and *ob/ob* (OB) mouse hearts (4 biological replicates per condition) (see Excel attached)**

**Supplemental table 4: Panther enrichment test (Bonferroni)**

| GO biological process | Number of proteins | Up + or Down - regulated in *ob/ob* | P value |
| --- | --- | --- | --- |
| regulation of transport (GO:0051049) | **150** | **+** | **0.0008** |
| regulation of localization (GO:0032879) | **186** | **+** | **0.0008** |
| regulation of system process (GO:0044057) | **59** | **+** | **0.0015** |
| mitochondrion organization (GO:0007005) | **126** | **-** | **0.0020** |
| vesicle-mediated transport (GO:0016192) | **75** | **+** | **0.0028** |
| regulation of heart contraction (GO:0008016) | **34** | **+** | **0.0032** |
| regulation of actin filament-based process (GO:0032970) | **34** | **+** | **0.0050** |
| multicellular organismal process (GO:0032501) | **248** | **+** | **0.0074** |
| positive regulation of biological process (GO:0048518) | **240** | **+** | **0.0100** |
| regulation of cytoskeleton organization (GO:0051493) | **27** | **+** | **0.0120** |
| regulation of blood circulation (GO:1903522) | **38** | **+** | **0.0121** |
| positive regulation of cellular process (GO:0048522) | **218** | **+** | **0.0129** |
| regulation of ion transport (GO:0043269) | **60** | **+** | **0.0131** |
| cellular response to stimulus (GO:0051716) | **201** | **+** | **0.0153** |
| regulation of supramolecular fiber organization (GO:1902903) | **26** | **+** | **0.0154** |
| regulation of protein-containing complex assembly (GO:0043254) | **28** | **+** | **0.0160** |
| animal organ development (GO:0048513) | **119** | **+** | **0.0170** |
| regulation of multicellular organismal process (GO:0051239) | **151** | **+** | **0.0200** |
| response to stimulus (GO:0050896) | **273** | **+** | **0.0211** |
| dicarboxylic acid metabolic process (GO:0043648) | **31** | **-** | **0.0233** |
| positive regulation of transport (GO:0051050) | **88** | **+** | **0.0244** |
| cell cycle (GO:0007049) | **31** | **+** | **0.0294** |
| regulation of cellular component biogenesis (GO:0044087) | **47** | **+** | **0.0294** |
| regulation of biological quality (GO:0065008) | **244** | **+** | **0.0329** |
| cell communication (GO:0007154) | **124** | **+** | **0.0446** |
| developmental process (GO:0032502) | **221** | **+** | **0.0455** |

**Supplemental Table 5: Dataset used to perform the principal component analysis**

**Supplemental Table 6: Percentage of contribution of each variable to the dimensions of biplot from Supplemental Figure 1.**

|  | Dim.1 | Dim.2 |
| --- | --- | --- |
| Body_Weight | 7.080479 | 1.611444 |
| E_A | 2.099627 | 10.85443 |
| IVRT | 5.132267 | 6.759356 |
| FS | 5.744074 | 0.028664 |
| LV_mass | 4.564141 | 9.982996 |
| PWD_thickness | 7.51408 | 2.629386 |
| PW_SR | 8.324381 | 0.477958 |
| AW_SR | 6.141618 | 0.005769 |
| Insulin_stimulated_P_AKT | 6.57164 | 3.630686 |
| Glycemia_fasted | 4.970005 | 5.401644 |
| MAM_Mito | 2.81484 | 14.57767 |
| Histamine_Peak_Amplitude | 7.062166 | 0.45008 |
| Resting_mito_Ca | 3.280414 | 0.166286 |
| Fibrosis | 2.913914 | 3.741439 |
| TG | 5.65898 | 7.257343 |
| ATP_content | 4.901519 | 6.938909 |
| MitoSOX | 3.985919 | 11.96038 |
| Shortening | 5.166368 | 7.000261 |
| Capacitance | 6.073567 | 6.525298 |

**Supplemental Figure 1: Uncropped membranes of Figure 1C**


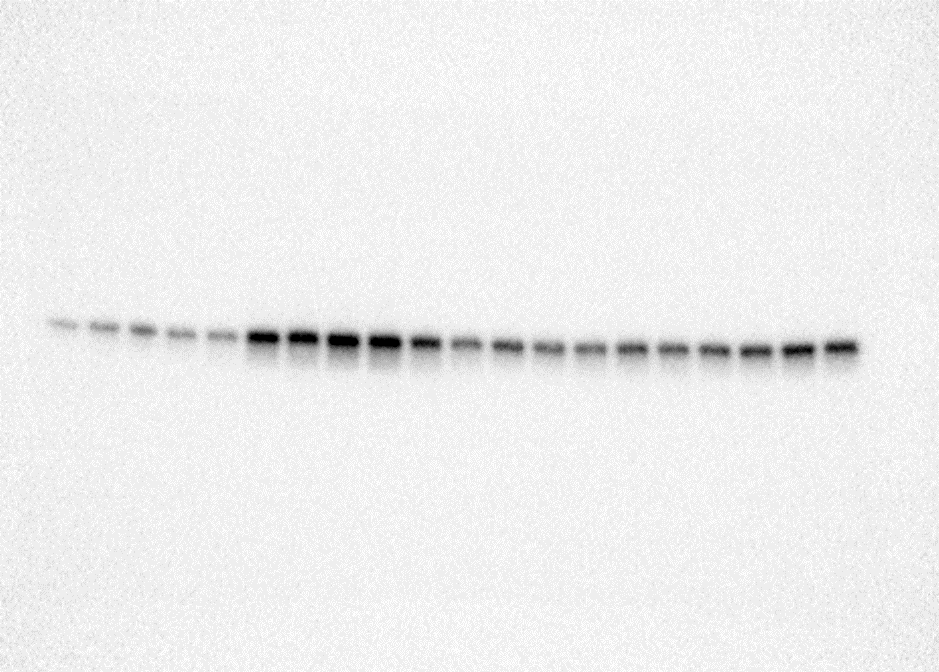


**70kDa**

**55kDa**

**pAKT**


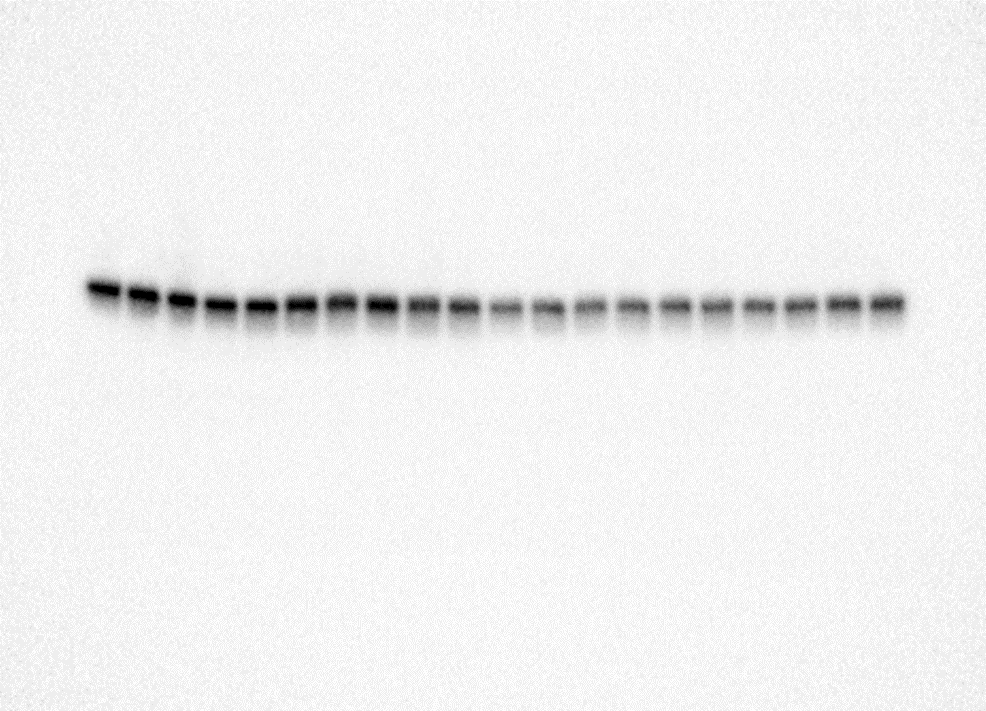


**70kDa**

**55kDa**

**AKT**

**Supplemental Figure 2: Uncropped membranes of Figure 2E**

**Supplemental Figure 3: Representative immunoblots of ER stress markers (A) and autophay (B) in cardiac lysates from WT and ob/ob mice.** Uncropped merged membranes. Grp78 (sc1050, 1/5000); peIF2a (Cell Signaling 9721, 1/1000); eIF2a (Cell Signaling 9722, 1/1000); CHOP (Cell Signaling 2895, 1/1000); pAMPK (Cell Signaling 2531, 1/250); AMPK (ab80039, 1/1000); LC3 (Cell Signaling 2772, 1/1000).

**B**

**A**

**Supplemental Figure 4: Biplot of the 4 groups with all the variables**
